# Uncontrolled blood pressure and associated factors in adult hypertensive patients undergoing follow-up at public health facility ambulatory clinics in Bishoftu town, Ethiopia: a multi-center study

**DOI:** 10.1186/s12872-023-03290-z

**Published:** 2023-05-17

**Authors:** Menawork Solomon, Yohannes Mekuria Negussie, Nardos Tilahun Bekele, Mihiret Shawel Getahun, Abenet Menene Gurara

**Affiliations:** 1 Department of Public Health, Adama Hospital Medical College, Adama, Ethiopia; 2 Department of Medicine, Adama General Hospital and Medical College, Adama, Ethiopia; 3 Department of Nursing, Adama General Hospital and Medical College, Adama, Ethiopia; 4 Department of Nursing, Arsi University, Asella, Ethiopia

**Keywords:** Uncontrolled blood pressure, Hypertension, Bishoftu, Ethiopia

## Abstract

**Background:**

Uncontrolled blood pressure is an important medical and public health problem in developing countries like Ethiopia. Improving the management of hypertension requires a better comprehension of the factors influencing blood pressure control and the application of interventions. But in clinical practice, blood pressure is still not adequately controlled. Thus, this study aimed to assess uncontrolled blood pressure and associated among adult hypertensive patients on follow-up at public health facility ambulatory clinics in Bishoftu, Ethiopia.

**Methods:**

A hospital-based cross-sectional study was conducted among 398 adult hypertensive patients who were on treatment and follow-up from April to May 31, 2022. Systematic random sampling was used to select study participants. Data were collected using an interviewer-administered, semi-structured questionnaire and chart review. The Eighth Joint National Committee (JNC 8) criteria was applied to define blood pressure control status. Binary logistic regression analysis was used to model the association between dependent and independent variables. An adjusted odds ratio and 95% confidence interval were used to measure the strength of the association. Finally, at a *p*-value < 0.05, statistical significance was proclaimed.

**Result:**

Of the total study participants, 249(62.6%) were male. The mean age was 62.26 ± 11.55 years. The overall proportion of uncontrolled blood pressure was 58.8% (95% CI: 54–64). Salt intake (AOR = 2.51; 95% CI: 1.49–4.24), lack of physical activity (AOR = 1.40; 95% CI: 1.10–2.62), habitual coffee consumption (AOR = 4.52; 95% CI: 2.67–7.64), higher BMI (AOR = 2.08; 95% CI: 1.24–3.49), and non-adherence to antihypertensive medications (AOR = 2.31; 95% CI: 1.3–3.89) were independent predictors of uncontrolled blood pressure.

**Conclusion:**

More than half of the hypertensive patients in this study had uncontrolled blood pressure. Healthcare providers and other accountable stakeholders should urge patients to follow salt restriction, physical activity, and antihypertensive medication regimes. Reduced coffee consumption and weight maintenance are other crucial blood pressure control measures.

## Background

Hypertension (HTN), the underlying cause of numerous bodily system and organ failures, continues to be a major global concern and public health issue [1, 2]. It was always thought to be a disease of developed nations, but its occurrence has shifted dramatically in recent decades, with Africa now outnumbering developed countries [3]. The persistent high burden of non-communicable disease, notably HTN, in the majority of low- and middle-income nations poses a serious public health concern nowadays [4].

Hypertension affects almost a billion people globally, with two-thirds of those people living in low-income nations [5]. One-third of adults globally have hypertension, and by 2025, this figure is expected to climb by 29% to 1.56 billion, with more than 125 million of those people residing in Sub-Saharan Africa [6, 7]. According to a systematic review and meta-analysis done in 2020, the prevalence of hypertension in Ethiopia is 21.81% [8].

Controlling HTN within a targeted blood pressure (BP) goal is crucial for lowering related morbidity and mortality. BP is deemed controlled when it is less than 150/90 mmHg with regular use of antihypertensive medication(s) for individuals aged 60 years and above and less than 140/90 mmHg in patients younger than the age of 60 years and all ages of hypertensive patients with diabetes and/or chronic kidney disease [9]. Despite the availability of several effective antihypertensive medications with proven advantages in lowering morbidity and mortality rates, hypertension remains poorly controlled in clinical practice [10, 11]. In developed countries, less than 27% of patients with HTN and less than 10% of patients with HTN in developing countries have been able to successfully control their BP. In Ethiopia, it is estimated that 48% of HTN patients had uncontrolled BP [12].

Patients with uncontrolled BP have a higher risk of heart failure and mortality from cardiovascular complications of HTN [13]. Long-term complications such as myocardial infarction, stroke, and renal disease are also significantly increased [11, 14]. The risk of severe cardiovascular and stroke incidents doubles for every 20 mmHg increase in SBP to > 115 mmHg or 10 mmHg increase in DBP to > 75 mmHg [11].

Globally, little is known about how well hypertensive patients control their BP and what factors contribute to poor BP control. According to previous studies, patients with HTN who have uncontrolled BP have a variety of contributing factors. Sex, age, occupation, duration since hypertension diagnosis, poor medication adherence, lack of exercise, salt intake, being overweight or obese, presence of comorbidity, and alcohol consumption were all linked to poor BP control [15–21].

The prevention of complications from HTN greatly depends on the recognition of the problems in BP control and the implementation of solutions [15, 16, 20]. Therefore, understanding the level of BP control in hypertensive patients will not only help healthcare providers manage hypertension effectively, but it can also help policymakers develop pertinent, context-specific policies that can enhance the management of hypertension.

The present study involved both hospitals and primary healthcare facilities, but most prior studies were conducted in one of these settings. While several studies have shown the magnitude and correlates of HTN, findings on the magnitude and factors that influence BP control among hypertensive patients on treatment and follow-up are lacking. Furthermore, the factors that contribute to uncontrolled BP are inconsistent across studies, and no study was conducted in the study setting among HTN patients to assess their BP control status. Thus, this study aimed to determine the level of uncontrolled BP and associated among adult hypertensive patients on follow-up at public health facility ambulatory clinics in Bishoftu town, Ethiopia.

## Methods and materials

### Study design, setting and period

A hospital-based cross-sectional study was conducted from April to May 31, 2022, in Bishoftu town. Bishoftu town is 47 km away from Addis Ababa and divided into 14 administrative kebeles. As of the 2007 national census, Bishoftu had a total population of 99,928 (47,860 men and 52,068 women). There are six health centers and two hospitals in the town. The current study was conducted in four health centers and two hospitals, namely Bishoftu Health Center, Cheleleka Health Center, Babogaya Health Center, Keta Health Center, Air Force Hospital (AFH), and Bishoftu General Hospital (BGH).

### Population

The source population was all patients with hypertension who were on follow-up at public health facility ambulatory clinics in Bishoftu town. The study populations were all hypertensive patients who were on follow-up at selected public health facility ambulatory clinics in Bishoftu town during the study period.

#### Inclusion criteria

All hypertensive patients over the age of 18 who had been on follow-up at the selected public health facilities for at least 6 months and came to the outpatient department of medicine for follow-up visits during the study period.

#### Exclusion criteria

Hypertensive pregnant women, patients with cognitive impairment, newly diagnosed patients, patients with hypertensive urgency or emergency, patients who were seriously ill at the time of data collection and unable to respond to the questions, patients who declined to participate in the study, and those whose medical records lacked information on their demographics and blood pressure were all excluded.

### Sample size determination and sampling procedure

The sample size was calculated using single population proportion formula with the assumptions of the magnitude of uncontrolled hypertension ( *p* = 0.37) taken from a previous study done in Nekemte Referral Hospital [19], 95% confidence level (the critical value Zα/2 = 1.96), and 5% margin of error and 10% contingency for non-response. Then, the final sample size becomes 398

$$n=\frac{{(z\alpha / 2)}^{2}p\left(1-p\right)}{{d}^{2}}$$

where: ***n*** = the required sample size, **Z α/2** = the standardized normal distribution curve value for the 95% confidence interval, ***p*** = the proportion of uncontrolled blood pressure, and **d** = the margin of error between the sample and population.

**Fig. 1:**
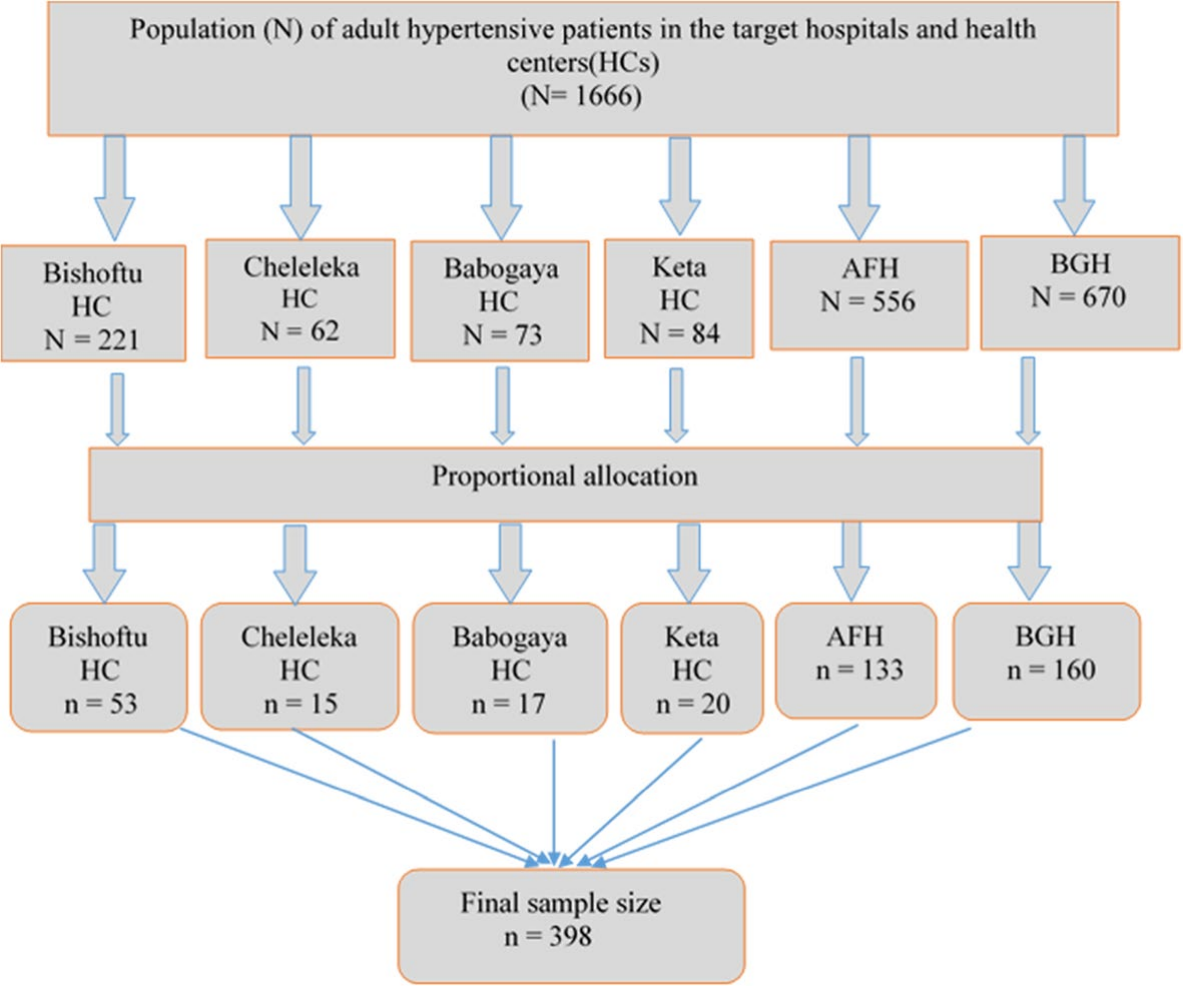
Schematic presentation of the sampling technique used to select the study participants from public health facilities of Bishoftu, Ethiopia, 2022

Health facilities were stratified by health centers and hospitals. From six health centers, four were selected by a simple random sampling method, and the two governmental hospitals were selected purposively. The calculated sample size was proportionally assigned to the chosen health facilities based on their previous three-month average client flow for hypertension follow-up during the study period. The study participants were drawn from each selected health facility using systematic random sampling. The sampling interval at each health facility was calculated by dividing the average number of hypertensive patients in the previous three months at selected health facilities by the required sample size to determine the required number of participants from each facility **(**Fig. 1).

### Study variables

#### Dependent variable

Uncontrolled blood pressure.

### Independent variables

#### Socio-demographic variables

Age, sex, marital status, place of residence, educational level, work status, and monthly income.

#### Clinical/Disease-related factors

Family history of hypertension, presence of comorbidity, duration of the disease, frequency of follow-up, frequency of BP measurement, follow-up miss, and body mass index (BMI).

#### Behavioral/Lifestyle-related factors

Smoking, alcohol drinking status, khat chewing, physical exercise, eating vegetables and fruit, salt intake, and habitual coffee consumption.

#### Medication-related factors

Duration of anti-hypertensive treatment, adherence to anti-hypertensive drugs, treatment modification, side effects, class of anti-hypertensive drugs, and the number of medications.

### Operational definitions and study measurements

#### Uncontrolled BP

Defined as Systolic blood pressure of ≥ 150 mmHg and/or Diastolic blood pressure of ≥ 90 mmHg with regular use of antihypertensive medication(s) for individuals age 60 years and above and Systolic blood pressure of ≥ 140 mm Hg and/or diastolic blood pressure of ≥ 90 mmHg) in patients younger than the age of 60 years and all ages of hypertensive patients with diabetes and/or chronic kidney disease [9].

BP measurements made by a physician who monitored hypertensive patients during their last six months follow-up visits were reviewed in the charts of adult hypertensive patients. The last BP was measured using a pretested standard mercury sphygmomanometer by trained data collectors. BP cuff with the proper cuff size, which covered two-thirds of the upper arm, while the patient was in a sitting position with 90-degree back support, rested for no less than five minutes, and avoided caffeine and cigarettes for 30 min before the measurement. Extra clothing that might interfere with the BP cuff has been removed. With a bare arm and the middle of the cuff at heart level, and uncrossed legs, without talking during or between measurements. During the measurement, the patients were kept relaxed. The BP cuff was inflated to the point where no noises were detected through the stethoscope. The cuff is then progressively deflated to measure the systolic and diastolic blood pressures. Two measurements were performed back-to-back with a minimum 5-minute gap, and the mean BP was recorded [9, 22, 23].

#### Medication adherence

Medication adherence was assessed with the Hill-Bone Compliance to High Blood Pressure Therapy Scale (HB-HBP) medication adherence subscale. The subscale has nine items with a four-point Likert-type scale: "none of the time," "some of the time," "most of the time," and "all of the time." The total scores range from 9 to 36, with higher scores reflecting poorer adherence to antihypertensive medication. Patients were deemed to be adherent if their scores were at or above the median [24–26].

#### Smoking

Smoking was assessed by one item whether the patients smoked or even a puff in the last seven days. Patients were classified as non-smokers if they had not smoked or had not taken even one puff in the last 7 days [27].

#### Alcohol consumption

Male patients who drank more than 2 units of alcohol per day and female patients who drank more than 1 unit of alcohol per day were considered alcohol consumers [15, 19].

#### Fruit and vegetable eating habits of patients

Were assessed and grouped according to the number of days they ate each per week into three: those who ate for less than one day per week (none), 1–3 days per week, and 4–7 days per week [15].

#### Habitual coffee consumption

Information on habitual coffee intake was collected with 1 item: "How many days of the week do you drink coffee?" Participants were defined as "coffee drinkers" if they drank coffee three or more times per week; otherwise, they were regarded as "non-drinkers" [28].

#### Physical activity

Physical activity was assessed by asking how many minutes per day and days per week a patient spent doing physical activity. This physical activity includes jobs or sports that require moderately intense activity that only slightly increases breathing or heart rate, such as cycling, swimming, volleyball, dancing, farming, gardening, and housework. It also includes activities like brisk walking, carrying light loads, swimming, and dancing. If the participants engaged in physical activity for at least 30 min per day, at least five days a week (≥ 150 min per week), they were considered physically active [15, 19, 29, 30].

#### Salt intake

Twelve items were used to measure the low-salt diet. After the mean was calculated, a patient is considered adherent to the low salt diet if they scored ≥ 6(indicating the participant followed low salt diet practice on 6 out of 7 days) [27].

### Data collection procedure and quality control

Data were collected using a semi-structured interviewer-administered questionnaire and document review. The questionnaires were adapted from validated scales and published articles and modified for the study's context [15, 18, 19, 27, 31, 32]. Data abstraction format was used to retrieve the necessary information from patients’ records. Patients were interviewed to obtain sociodemographic, disease-related, lifestyle-related, and medication-related information. Three trained nurses collected data from various facilities while being supervised by two public health officers.

The English version of the questionnaire was prepared. Then it was translated into Amharic and Afan oromo versions (local languages) and back to English to ensure consistency. To ensure the quality and consistency of the data abstraction format and the questionnaire with the objective of the study, a pre-test was done on 5% of the total sample of patients (*n* = 20) at non-selected public health facilities, and a correction was made accordingly. Two days of training were organized for data collectors and supervisors on the procedure of data collection and the purpose of the study. All collected data were examined for completeness and consistency during data management, storage, and analysis.

### Data processing and analysis

Data were coded and entered into Epi-Info Version 7 and exported to the Statistical Package for Social Sciences (SPSS) Version 26 for cleaning and statistical analysis. Normality assumptions for continuous variables were checked using the Shapiro–Wilk test. To explain the study population with relevant variables, descriptive statistics were used. Binary logistic regression analysis was used to model the associations between uncontrolled blood pressure and independent variables. The statistical assumptions for binary logistic regression, i.e., multi-collinearity, normality, linearity, independence of residuals, and outliers, were assessed, and no significant violations were identified. The regression model was fitted using the standard model-building approach. The goodness of fit of the final model was checked using the Hosmer and Lemeshow test, and the result was significant with a *p*-value > 0.05. In the bi-variable logistic regression model, a *p*-value of < 0.25 was used as a cut-off value to select variables for multivariable logistic regression analysis to control the potential effects of confounders. In the final model, the adjusted odds ratio (AOR) with a 95% confidence interval (CI) was used to determine predictors of uncontrolled blood pressure. At this level, a *p*-value of < 0.05 was considered statistically significant.

## Result

### Socio-demographic characteristics

A total of 398 adult hypertensive respondents were interviewed, making up a 100% response rate. The mean age of the participants was 62.26 (SD: ± 11.55) years, with a minimum age of 35 and a maximum age of 89 years. Two hundred forty-nine (62.6%) of the respondents were male, 282 (70.9%) were married, and 328 (82.4%) were urban residents. The majority of them, 147 (36.6%), attended secondary school, and 164 (41.2%) had a monthly income between 2000 and 4000 Birr (Table 1).

**Table 1:** Socio-demographic characteristics of hypertensive patients on follow-up at ambulatory clinics of public health facilities in Bishoftu town, Ethiopia, 2022 (*n* = 398)

Variables	Category	Frequency	Percent
**Age (years)**	< 65	244	61.3
	≥ 65	154	38.7
**Sex**	Male	249	62.6
	Female	149	37.4
**Marital status**	Single	23	5.8
	Married	282	70.9
	Divorced	25	6.3
	Widowed	68	17.1
**Place of residence**	Urban	328	82.4
	Rural	70	17.6
**Educational level**	No formal education	88	22.1
	Primary school (1–8 grade)	50	12.6
	Secondary school (9–12 grade)	147	36.9
	College and above	113	28.4
**Work status**	Farmer	43	10.8
	Government employee	85	21.4
	Housewife	74	18.6
	Merchant	51	12.8
	Retired	141	35.4
	Others*	4	1
**Family monthly income (ETB)**	< 2000	95	23.9
	2000–4000	164	41.2
	4001–6000	74	18.6
	> 6000	65	16.3

Notes: * Daily labor, and Non-governmental organization employee

Abbreviation: *ETB* Ethiopian birr

### Clinical/Disease-related characteristics

In this study, 127 (31.9%) participants had a family history of HTN, and 148 (37.2%) of them were diagnosed with HTN between 5 and 10 years. Of the participants, 171 (43%) had a history of missed follow-up, and 195 (49%) measured their BP monthly. About 152 (38.2%) had a comorbid illness, and of those, 44.2% had diabetes. Moreover, 188 (47.2%) had BMI measurements within the normal range (Table 2).

**Table 2:** Clinical/Disease-related characteristics of hypertensive patients on follow-up at ambulatory clinics of public health facilities in Bishoftu town, Ethiopia, 2022 (*n* = 398)

Variable	Category	Frequency	Percent
**Family history of HTN**	Yes	127	31.9
	No	271	68.1
**Duration of HTN diagnosis (years)**	< 5	129	32.4
	5–10	148	37.2
	> 10	121	30.4
**Frequency of follow-up**	Monthly	316	79.4
	Every two month	80	20.1
	Other*	2	0.5
**Follow-up miss**	Yes	171	43
	No	227	57
**Frequency of BP measurement**	Every two month	49	12.3
	Every two weeks	88	22.1
	Monthly	195	49
	Weekly	66	16.6
**Comorbidity**	Yes	152	38.2
	No	246	61.8
**Types of comorbidities (*n* = 152)**	Diabetes	79	44.2
	Chronic kidney disease	23	12.9
	Myocardial infarction	45	25.2
	Stroke	23	12.6
	Hyperlipidemia	9	5.1
**Body mass index (kg/m**^**2**^**)**	Normal (18.5–24.99)	181	45.5
	Overweight (25–29.9)	188	47.2
	Obese (≥ 30)	29	7.3

Notes: * Every two week

Abbreviations: *BP* Blood pressure, *HTN* Hypertension

### Behavioral/Life Style-Related Characteristics

In this study, only 16 (4%) were current smokers, and 184 (246.2%) were habitual coffee drinkers. Among the study participants, 253 (63.6%) did not eat vegetables, and 277 (69.6%) did not eat fruits 1–3 days per week (Table 3).

**Table 3:** Lifestyle-related characteristics of hypertensive patients on follow-up at ambulatory clinics of public health facilities in Bishoftu town, Ethiopia, 2022 (*n* = 398)

Variable	Category	Frequency	Percent
**Smoking**	Yes	16	4
	No	382	96
**Alcohol consumption**	Yes	48	12.1
	No	350	87.9
**Khat** **chewing**	Yes	9	2.3
	No	389	97.7
**Habitual coffee consumption**	Yes	184	46.2
	No	214	53.8
**Vegetable eating habit**	1–3 days/week	253	63.6
	4–7 days/week	102	25.6
	None	43	10.8
**Fruit-eating habit**	1–3 days/week	277	69.6
	4–7 days/week	11	2.8
	None	110	27.6
**Salt intake**	Yes	205	51.5
	No	193	48.5
**Physical activity**	Yes	158	39.7
	No	240	60.3

### Medication-related characteristics

Among the participants, 191 (48%) received their medication free of charge, and 109 (27.4%) have been on antihypertensives for more than ten years. About 130 (32.7%) participants reported experiencing side effects, and weakness was the most common side effect due to medications. Thiazide diuretics were the most widely used anti-hypertensive medications, comprising 36.7% (Table 4).

**Table 4:** Medication-related characteristics of hypertensive patients on follow-up at ambulatory clinics of public health facilities in Bishoftu town, Ethiopia, 2022 (*n* = 398)

Variable	Category	Frequency	Percent
**Source of medication**	By sponsorship	83	20.9
	Free of charge	191	48
	Self-sponsored	124	31.1
**Duration of therapy (years)**	< 5	142	35.7
	5–10	147	36.9
	> 10	109	27.4
**Side effect**	Yes	130	32.7
	No	268	67.3
**Experienced side effects (*n* = 130)**	Dry mouth	27	20.8
	Erectile dysfunction	16	12.3
	Headache	27	20.8
	Weakness	59	45.4
	Other*	1	0.7
**Class of drugs**	ACE inhibitors	95	23.9
	Calcium channel blockers	127	31.9
	Thiazide diuretics	146	36.7
	Beta-blockers	30	7.5
**Number of medications**	Monotherapy	286	71.9
	Drug combination	112	28.1
**Medication adherence**	Adherent	175	44
	Non-adherent	223	56

Notes: * Edema

Abbreviations: *ACE* Angiotensin-converting enzyme

### The magnitude of uncontrolled blood pressure

**Fig. 2:**
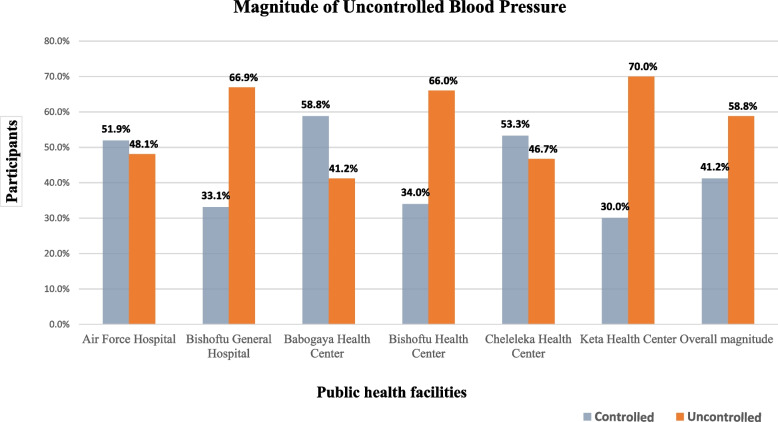
Blood pressure control status among hypertensive patients on follow-up at ambulatory clinics of public health facilities in Bishoftu town, Ethiopia, 2022

The result of this study showed that the overall magnitude of uncontrolled blood pressure among hypertensive patients in public health facilities in Bishoftu town was 58.8% (95% CI: 54, 64). The highest and lowest levels of uncontrolled blood pressure were found in Keta and Babogaya Health Centers, respectively (Fig. 2)**.**

### Factors associated with uncontrolled blood pressure

After the model has been checked for multi-collinearity and model fitness; variables with a *p*-value < 0.25 in the bivariate analysis were chosen as candidate variables for the final model. Hence, in the bivariate analysis, age, family history of hypertension, duration of HTN diagnosis, higher BMI, habitual coffee consumption, salt intake, lack of physical activity, duration of therapy, number of medications, and non-adherence to antihypertensive medications showed statistically significant associations with uncontrolled BP. Following adjustment for potential confounding factors with multivariable binary logistic regression analysis, salt intake, lack of physical activity, habitual coffee consumption, higher BMI, and non-adherence to antihypertensive medications were independent predictors of uncontrolled BP at a *p*-value < 0.05.

Hypertensive patients who added salt to their food had 2.5 times greater odds of uncontrolled BP compared to hypertensive patients who used no salt in their food (AOR = 2.51, 95% CI: 1.49–4.24). The odds of uncontrolled BP among hypertensive patients not involved in physical activity were 1.4 times greater compared to hypertensive patients involved in physical activity (AOR = 1.4, 95% CI: 1.10–2.62). Compared to non-coffee-drinking hypertensive patients, those hypertensive patients who were habitual coffee consumers had 4.5 times the odds of having uncontrolled BP (AOR = 4.52, 95% CI: 2.67–7.64). Hypertensive patients with higher BMI (overweight and/or obese) had two times greater odds of uncontrolled BP compared to those who were normal (AOR = 2.08, 95% CI: 1.24–3.49). Furthermore, patients who were non-adherent to their anti-hypertensive drugs had 2.3 times greater odds of uncontrolled BP than those who were adherent (AOR = 2.31, 95% CI: 1.37–3.89) (Table 5).

**Table 5:** Factors associated with uncontrolled blood pressure among hypertensive on follow-up at ambulatory clinics of public health facilities in Bishoftu town, Ethiopia, 2022

Variable	Category	Blood pressure control status		COR (95% CI)	AOR (95% CI)
		**Controlled**	**Uncontrolled**		
**Age (years)**	< 65	124	120	1	1
	> 65	40	114	2.9(1.89,4.57) *	1.4(0.73,2.75)
**Family history**	Yes	66	61	1	1
	No	98	173	0.5(0.34,0.80) *	1.2(0.64,2.09)
**Duration of HTN diagnosis**	< 5	72	70	1	1
	5–10	59	88	1.5(0.95,2.47)	0.8(0.18,3.48)
	> 10	33	76	2.3(1.37,3.83) *	0.5(0.06,3.46)
**Body mass index (k/m**^**2**^**)**	18.5–24.9	100	81	1	1
	> 25	64	153	3.0(2.00,4.62) *	2.08(1.24,3.49) **
**Habitual coffee consumption**	No	56	158	1	1
	Yes	108	76	4.0(2.63,6.12) *	4.52(2.67,7.64) **
**Salt intake**	No	60	145	1	1
	Yes	104	89	2.8(1.86,4.26) *	2.51(1.49,4.24) **
**Physical activity**	No	65	175	0.22(0.10,0.92) *	1.4(1.10,2.62) **
	Yes	99	59	1	1
**Duration of therapy**	< 5 years	72	70	1	1
	5–10 years	59	88	1.5(0.96,2.44)	1.2(0.27,5.07)
	> 10 years	33	76	2.4(1.40,4.0) *	1.6(0.22,11.07)
**Medications**	Monotherapy	131	155	1	1
	Drug combination	33	79	2.0(1.27,3.23) *	0.8(0.41,1.65)
**Medication adherence**	Adherent	102	73	1	1
	Non-adherent	62	160	3.6(2.37,5.49) *	2.31(1.37,3.89) **

Notes: Significant at p-value < 0.25 in unadjusted logistic regression analysis, **significant at p < 0.05 in adjusted logistic regression analysis, 1 = Reference

Abbreviations: *COR* Crude odds ratio, *CI* Confidence interval, *AOR* Adjusted odds ratio

## Discussion

In hypertensive patients, maintaining proper blood pressure control leads to favorable therapeutic outcomes, such as a reduction in HTN-related mortality and complications, which lowers the disease's global toll. Studies have shown that a significant majority of hypertensive patients still have uncontrolled BP despite receiving treatment [31, 33]. This study revealed that the overall magnitude of uncontrolled BP in Bishoftu town public health facilities was relatively high. The multivariable logistic regression analysis showed that salt intake, lack of physical activity, habitual coffee consumption, higher BMI, and non-adherence to antihypertensive medications were all independent predictors of uncontrolled BP.

This study showed that the magnitude of uncontrolled BP was found to be 58.8% (95% CI: 54˗64%). This is in line with studies conducted in Tikur Anbesa General Specialized Hospital, Addis Ababa (59.9%) [34], Debre Tabor District Hospital, Northwest Ethiopia (57.1%) [20], Bale Zone Public Hospitals (56.7%) [35], Bedele General Hospital, Southwest Ethiopia (56.2%) [17], Public Health Facilities in Dessie City, Northeast Ethiopia (55.8%) [36], hospitals in Yaoundé, Cameroon (63.2%) [37], and a national survey in Vietnam (54.1%) [38]. However, it was higher than studies done at Nekemte Referral Hospital, Western Ethiopia (36.4%) [19], studies done at the University of Gondar Referral Hospital, Northwest Ethiopia (37% and 49.6%) [15, 39], a primary care facility in South Africa (43%) [40] and studies conducted in Thailand [41], Chile [29], and Spain [42], which revealed uncontrolled BP of 24.6%, 36.9%, and 44.6%, respectively. Moreover, the magnitude of uncontrolled BP in this study was lower than in two studies done in Zewditu Memorial Hospital, Addis Ababa (73.8% and 69.9%) [2, 43], and studies in Regional Referral Hospital, Kenya [44], and Jordan [45], which showed 66.6% and 67.1% of uncontrolled BP, respectively.

These differences could be explained by the fact that in our study, the majority of the patients had regular follow-up visits at a dedicated health facility, as well as the difference in study design and sample size. Furthermore, the disparity could be attributed to sociocultural and behavioral differences among the study population as well as differences in the expertise of healthcare professionals involved in the management of HTN and healthcare services in the study settings. In addition, the inconsistency may also be linked to differences in antihypertensive drug adherence rates and variations in the criteria utilized to classify hypertensive patients as having uncontrolled or controlled BP. Most studies used the JNC7 guideline [14], which employed a cutoff value of > 140/90 for non-diabetic patients and > 130/80 for diabetic patients to define uncontrolled BP, and Some studies applied the European Society of Cardiology/European Society of Hypertension (ESC/ESH) [46] and the American College of Cardiology/American Heart Association (ACC/AHA) [47] guidelines, which both lowered BP targets to 130/80 mmHg. Still, others utilized the new National Institute for Health and Care Excellence (NICE) guideline [48], which still suggests a threshold and target blood pressure of 140/90 mmHg, but the current study followed the JNC8 guideline [9].

In this study, hypertensive patients who added salt to their food had 2.5 times higher odds of uncontrolled BP compared to hypertensive patients who used no salt in their food. This finding is consistent with studies from the University of Gondar Referral Hospital and Debre Tabor District Hospital in Northwest Ethiopia [15, 20], which found that patients who used top-added salt on a plate were less likely to have optimal BP control than patients who did not use top-added salt. Studies from Zimbabwe [49] and Southern China [50] have also shown an association between salt consumption and BP. This can be explained by the fact that salt affects the body's natural sodium balance, leading to fluid retention and raising the pressure imposed by the blood on blood vessel walls, resulting in high blood pressure [51].

Physical activity was another significant factor associated with uncontrolled BP. In this study, the odds of uncontrolled BP among hypertensive patients not involved in physical activity were 1.4 times greater compared to those of hypertensive patients involved in physical activity. This finding is similar to studies conducted at Ayder Comprehensive Specialized Hospital in Tigray, Ethiopia [16]; Nekemte Referral Hospital in Western Ethiopia [19]; and Southern China [50]. Patients who engaged in physical activity were more likely to have optimal BP control than those who did not, according to studies conducted at the University of Gondar Referral Hospital and Debre Tabor District Hospital in Northwest Ethiopia [15, 20]. This can be justified by the fact that regular exercise strengthens the heart, allowing it to pump more blood with less exertion. So, when the heart is working less to pump blood, the strain on the arteries lessens, reducing BP. Physical activity also lowers high BP by lowering body weight, boosting renal function, and lowering systemic vascular resistance (vasoconstriction regulation), plasma norepinephrine, insulin sensitivity, and renin activity [52, 53].

In this study, in comparison to non-coffee-drinking hypertensive patients, habitual coffee users had 4.5 times the odds of having uncontrolled blood pressure. This is similar to a study conducted in Spain [54] which showed habitual coffee consumption was statistically associated with uncontrolled BP in hypertensive patients. This may be due to Caffeine has been hypothesized to elevate blood pressure by several mechanisms, such as sympathetic overactivity, adenosine receptor antagonism, elevated norepinephrine release by direct effects on the adrenal medulla, renal effects, and renin-angiotensin system activation. Conversely, different studies have produced inconsistent findings about the relationship between blood pressure and coffee consumption [55, 56]. The nature of the association between coffee consumption and BP is still unclear, and further studies are required to establish the association between uncontrolled BP and habitual coffee consumption.

This study revealed that hypertensive patients who were overweight and/or obese had two times higher odds of uncontrolled BP compared to those who were normal. This finding is in agreement with studies done at Jimma University Teaching and Specialized Hospital, Ethiopia [21]; Ayder Comprehensive Specialized Hospital, Tigray, Ethiopia [16]; Zimbabwe [49]; and Southern China [50]. A study done at the University of Gondar Referral Hospital, Northwest Ethiopia [15], also found that the likelihood of BP control was reduced by 50% for overweight and 44% for obese patients compared with their counterparts. The justification could be that a higher BMI (overweight and obesity) causes a state of chronic volume overload because of the increased demands on the circulatory system to move blood through vast and comparatively low-resistance adipose tissue. The renin-angiotensin system, the proportion of intra-abdominal and intravascular fat, sodium retention that raises renal reabsorption, and the sympathetic nervous system are all thought to play essential roles in the etiology of obesity-related hypertension [57, 58].

Adherence to medications is critical for preventing the effects of HTN-related morbidities and mortality. One of the main causes of poor BP control is poor medication adherence, which also affects the efficacy of health outcomes broadly and dampens the optimal therapeutic values. In line with studies conducted in Nekemte Referral Hospital, Western Ethiopia [19]; Debre Tabor District Hospital, Northwest Ethiopia [20]; Ayder Comprehensive Specialized Hospital, Tigray, Ethiopia [16]; Yaoundé, Cameroon [37]; and Southern California, USA [59], this study showed patients who were non-adherent to their anti-hypertensive drugs were more likely to have uncontrolled BP than those who were adherent. This is because anti-hypertensive drugs decrease and manage high BP by boosting vasodilatation, reducing vasoconstriction, raising urine output, and preventing sympathetic heart activation [60] and good antihypertensive medication compliance is crucial for managing hypertension and lowering BP.

### Limitations of the study

Due to the cross-sectional study design used in this study, it is impossible to determine temporal relationships and difficult to confirm the causal-effect relationship between the dependent and predictor variables. The classes of drugs used and their impact on BP control was not investigated in detail. Moreover, there might be recall and social desirability biases since the study participants' behavioral practices were based on self-reports, and the performance of these behaviors was not observed or validated.

## Conclusion

In conclusion, the proportion of hypertensive patients with uncontrolled BP was relatively high in the study setting. Salt intake, lack of physical activity, habitual coffee consumption, higher BMI, and non-adherence to antihypertensive medications were factors associated with uncontrolled BP. Maintaining controlled BP is critical to avoiding the effects of uncontrolled BP. Healthcare providers and other responsible stakeholders should encourage patients to adhere to antihypertensive medications, physical activity, lifestyle modification, and adherence counseling measures to successfully control BP. Furthermore, studies using physical and biochemical markers to pinpoint the most significant factors linked to BP control, impediments to successful BP control, and treatment options to enhance treatment results are recommended.

## Data Availability

All data and materials are available from the corresponding author without undue reservation.

## Abbreviations

AFH: Air Force Hospital
AOR: Adjusted Odds Ratio
BGH: Bishoftu General Hospital
BMI: Body Mass Index
BP: Blood Pressure
COR: Crude Odds Ratio
CI: Confidence Interval
HTN: Hypertension
JNC: Joint National Commission
